# Hypogravity reduces trunk admittance and lumbar muscle activation in response to external perturbations

**DOI:** 10.1152/japplphysiol.00756.2019

**Published:** 2020-03-12

**Authors:** Enrico De Martino, Sauro E. Salomoni, Andrew Winnard, Kristofor McCarty, Kirsty Lindsay, Sherveen Riazati, Tobias Weber, Jonathan Scott, David A. Green, Julie Hides, Dorothée Debuse, Paul W. Hodges, Jaap H. van Dieën, Nick Caplan

**Affiliations:** ^1^Aerospace Medicine and Rehabilitation Laboratory, Faculty of Health and Life Sciences, Northumbria University, Newcastle upon Tyne, United Kingdom; ^2^NHMRC Centre for Clinical Research Excellence in Spinal Pain, Injury and Health, School of Health and Rehabilitation Sciences, The University of Queensland, Brisbane, Queensland, Australia; ^3^European Astronaut Centre, Space Medicine Team (HRE-OM), European Space Agency, Cologne, Germany; ^4^School of Allied Health Sciences, Griffith University, Nathan Campus, Brisbane, Queensland, Australia; ^5^Department of Human Movement Sciences, Vrije Universiteit Amsterdam, Amsterdam Movement Sciences, Amsterdam, The Netherlands; ^6^KBR, Wyle Laboratories GmbH, Cologne, Germany; ^7^Centre of Human and Applied Physiological Sciences, King’s College London, London, United Kingdom

**Keywords:** intramuscular electromyography, low gravity, lumbar spine, parabolic flight, trunk stabilization

## Abstract

Reduced paraspinal muscle size and flattening of spinal curvatures have been documented after spaceflight. Assessment of trunk adaptations to hypogravity can contribute to development of specific countermeasures. In this study, parabolic flights were used to investigate spinal curvature and muscle responses to hypogravity. Data from five trials at 0.25 g, 0.50 g, and 0.75 g were recorded from six participants positioned in a kneeling-seated position. During the first two trials, participants maintained a normal, upright posture. In the last three trials, small-amplitude perturbations were delivered in the anterior direction at the T_10_ level. Spinal curvature was estimated with motion capture cameras. Trunk displacement and contact force between the actuator and participant were recorded. Muscle activity responses were collected by intramuscular electromyography (iEMG) of the deep and superficial lumbar multifidus, iliocostalis lumborum, longissimus thoracis, quadratus lumborum, transversus abdominis, obliquus internus, and obliquus externus muscles. The root mean square iEMG and the average spinal angles were calculated. Trunk admittance and muscle responses to perturbations were calculated as closed-loop frequency-response functions. Compared with 0.75 g, 0.25 g resulted in lower activation of the longissimus thoracis (*P* = 0.002); lower responses of the superficial multifidus at low frequencies (*P* = 0.043); lower responses of the superficial multifidus (*P* = 0.029) and iliocostalis lumborum (*P* = 0.043); lower trunk admittance (*P* = 0.037) at intermediate frequencies; and stronger responses of the transversus abdominis at higher frequencies (*P* = 0.032). These findings indicate that exposure to hypogravity reduces trunk admittance, partially compensated by weaker stabilizing contributions of the paraspinal muscles and coinciding with an apparent increase of deep abdominal muscle activity.

**NEW & NOTEWORTHY** This study presents for the first time novel insights into the adaptations to hypogravity of spinal curvatures, trunk stiffness, and paraspinal muscle activity. We showed that exposure to hypogravity reduces the displacement of the trunk by an applied perturbation, partially compensated by weaker stabilizing contributions of the paraspinal muscles and concomitant increase in abdominal muscle responses. These findings may have relevance for future recommendations for planetary surface explorations.

## INTRODUCTION

The spine is affected by exposure to microgravity (42). During a space mission, the spinal column lengthens more than two times the average daily values (7, 48). The length change has been attributed to an increase of disk height beyond normal viscoelastic limits and to a reduction of the thoracic and lumbar curvatures (48). These morphological adaptations may weaken the annulus fibrosus, increasing the risk of herniated nucleus pulposus when gravity returns (21). Prolonged spinal unloading is also associated with trunk muscle atrophy, in particular the muscles that maintain an upright posture (25, 26). Studies using magnetic resonance imaging (MRI) have shown that long-duration spaceflight reduces paraspinal muscle cross-sectional area by ~8–9% at the L_3_–L_4_ vertebral level (3) and this reduction correlates with postflight decreases in lumbar lordosis (3). Impaired lumbar muscle function may increase the risk of traumatic stress of the intervertebral disk (IVD), in particular if the IVD is degenerated. In the absence of muscle contraction, buckling of the spine may cause IVD injuries, as these can occur with a rotation of as little as 2° in a healthy spine (15). However, to date only morphological evidence of muscle atrophy after long-term space missions is available (8, 27), and no studies have investigated the acute effect of transient reduction of gravity on lumbar muscle function. Investigation of the effects of transient exposure to different gravitational levels on the neuromuscular activity of trunk muscles will allow the identification of which muscles are most sensitive to the gravitation transitions involved in spaceflights. This knowledge is a first step toward understanding the impacts of exposure to this environment and for the development of tailored countermeasures to prevent back pain and spinal injury in astronauts.

Recently, a new framework has been developed to evaluate low-back stabilization by measuring trunk displacements around upright posture in response to unpredictable and completely known destabilizing perturbations delivered to the thorax (17, 43). The activity from paraspinal and abdominal muscles is also recorded with electromyography (EMG) and used to assess their contribution to trunk stabilization (30, 50, 52). Using this well-defined framework in a controlled environment of hypogravity could help researchers to understand the mechanisms and gravity dependencies of trunk stabilization, as well as identify which paraspinal and abdominal muscles are most affected.

The present study aimed to determine the acute effects of hypogravity at 0.25 g, 0.5 g, and 0.75 g on trunk admittance, which describes the trunk displacement as a function of contact force, and on the activity of the abdominal and paraspinal muscles at rest as well as during trunk perturbation. As hypogravity is expected to reduce the destabilizing effects of gravity on the trunk, we hypothesized that acute exposure to hypogravity would reduce trunk admittance during external perturbations in a dose-dependent manner. With the reduced requirement for stabilization, we further hypothesized that hypogravity would also decrease the contribution of paraspinal muscles and induce flattening of the lumbar lordosis during upright posture.

## MATERIALS AND METHODS

### 

#### Participants.

Six healthy volunteers (5 men, 1 woman; 41 ± 8 yr, 180 ± 9 cm, and 74 ± 12 kg) provided written informed consent to participate in the study, which received ethical approval from the Agence Française de Sécurité Sanitaire des Produits de Santé and the Northumbria University Institutional Review Board. The sample size was limited by the inherent restrictions associated with this single European Space Agency-funded flight parabolic campaign. Participants were pain-free at the time of testing and reported that they did not have a history of chronic musculoskeletal or other medical disorders that would affect the study. Participants received a subcutaneous injection of scopolamine hydrobromide (<0.25 mg/1 mL) to prevent motion sickness during the flight.

#### Study design.

Three parabolic flights were provided by NOVESPACE in Bordeaux-Mérignac Airport (Bordeaux, France). Each parabolic flight session comprised 31 parabolas, with a single familiarization parabola followed by three sets of 5 parabolas at 0.25 g, 0.5 g, and 0.75 g (total: 15 parabolas). These three sets of 5 parabolas were then repeated within the same flight session, allowing the assessment of two participants during each session. The sequence of parabolas during the first flight session was 0.25 g, 0.5 g, and 0.75 g (*day 1*); during the second flight session 0.5 g, 0.75 g, and 0.25 g (*day 2*); and during the third flight session 0.75 g, 0.25 g, and 0.5 g (*day 3*).

**Fig. 1.**
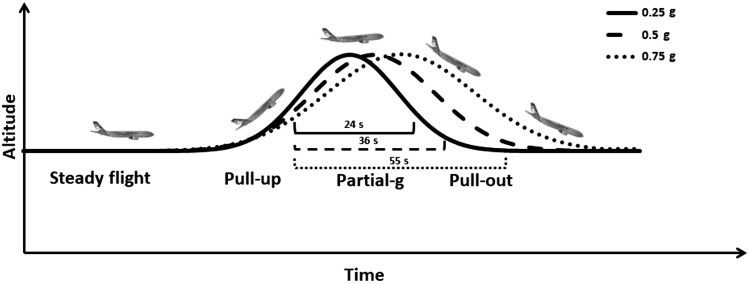
Schematic depicting the different phases of parabolic flight profile at each gravitational level (0.25 g, 0.50 g, and 0.75 g).

Each parabola comprised five time windows with distinct gravity conditions: level flight (1 g); hypergravity (~1.8 g) during the initial pull-up phase (15–22 s, depending on the target g level); hypogravity at 0.25 g, 0.5 g, or 0.75 g (24 s, 36 s, or 55 s, respectively); and a second period of hypergravity (~1.6–1.8 g) during the pull-out phase of the parabola (15–22 s, depending on the target g level), before returning to level flight at 1 (Fig. 1). All the analyses of this study focused on the periods of hypogravity.

Two participants were assessed during each parabolic flight; thus 15 parabolas per participant yielded 5 parabolas (trials) at each g level. During the first two experimental parabolas at each gravity level, participants were asked to relax while maintaining an upright posture with their arms by their sides (rest). During the subsequent three parabolas at each gravity level, participants were asked to resist a series of small-amplitude trunk perturbations controlled by a linear actuator (perturbation).

#### Rest: spinal curvature and muscle activity.

**Fig. 2.**
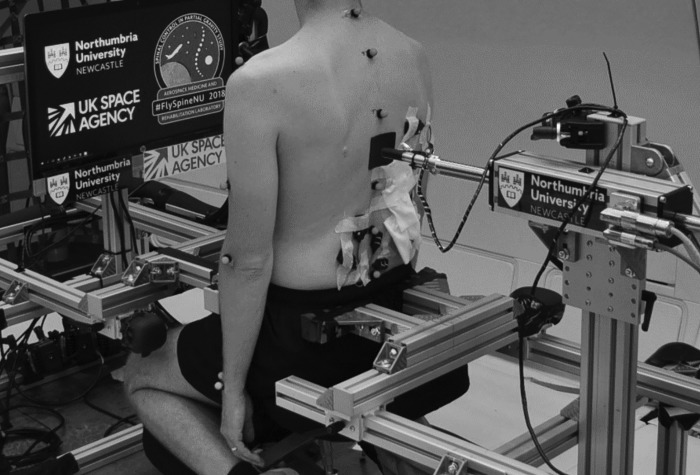
Experimental setup, showing the participant position and the linear actuator applying a posterior-anterior force to the participant’s trunk at T_10_. Note the 6 retroreflective markers positioned over the spinous processes of C_6_, T_3_, T_7_, T_12_, L_3_, and S_1_.

Participants assumed a kneeling-seated position. Restraints were placed below the anterior superior iliac spine and the posterior superior iliac spine to reduce pelvic motion. Participants were blindfolded and were instructed to maintain their head in an upright and consistent position to minimize the changes in contribution of the visual and vestibular information (other than that induced by acceleration due to gravity) to stabilization of the trunk (Fig. 2). Six reflective markers (diameter 14 mm) were attached with double-sided adhesive tape over the spinous processes of C_7_, T_3_, T_7_, T_12_, L_3_, and S_1_. A three-dimensional motion capture system with 14 opto-electronic cameras (Vertex; Vicon Motion Systems, Oxford, UK), controlled by Nexus (version 2.7) software, was used to record marker trajectories at a sampling rate of 200 Hz. The motion capture system was calibrated after takeoff with a standard dynamic protocol with a five-marker calibration wand. System calibration was accepted when the image error was <0.2 mm.

Spinal muscle activity was recorded with bipolar fine-wire intramuscular electromyography (iEMG) electrodes: two Teflon-coated 75-μm stainless steel wires with 1 mm of insulation removed from the ends, bent back to form hooks at 2- and 3-mm lengths and threaded into a hypodermic needle (22 gauge × 5.08 cm). On the right side, electrodes were inserted with ultrasound guidance (Logiq E BT12; General Electric, Duluth, MN) with a linear transducer (12L-RS; General Electric, Duluth, MN) into the deep lumbar multifidus (deep MF), superficial lumbar multifidus (superficial MF), iliocostalis lumborum pars lumborum (IL), longissimus thoracis pars thoracis (LO), quadratus lumborum (QL), transversus abdominis (TrA), obliquus internus (OI), and obliquus externus (OE) muscles on the right side of the trunk.

For deep MF, the needle was inserted ∼3 cm lateral to the L_4_ spinous process until the needle reached the most medial aspect of the lamina L_4_ (37). For superficial MF, the needle was inserted ~3 cm lateral to the L_4_ spinous process to ~10 mm below the skin surface (37). For IL, the needle was inserted ~8 cm lateral to the L_2_ spinous process ~10 mm below the skin surface (9). For LO, the needle was inserted ~4 cm lateral to the T_10_ spinous process directed toward the dorsal aspect of the transverse process (28). For QL, the needle was inserted ~10 cm lateral to the L_3_ spinous process near the muscle’s medial border (39). For OE, OI, and TrA, the needle was inserted midway between the anterior superior iliac spine (ASIS) and the rib cage into the belly of each muscle, with ~10 mm between insertion sites (19). After insertion, the hypodermic needles were removed, leaving only the wires in situ.

Each electrode was connected to a wireless EMG preamplifier (Trigno; Delsys, Boston, MA) with spring contact sensors. The sensors were attached to the skin of the participant with adhesive tape. EMG signals were preamplified (×100), transmitted telemetrically to a data receiver (Trigno Digital Base Station; Delsys, Boston, MA), band-pass filtered (25–1,000 Hz), sampled at 2,000 Hz (Lock+; Vicon Motion Systems, Oxford, UK), and stored for later analysis (Nexus 2.7; Vicon, Oxford, UK). During data analysis, EMG signals were digitally filtered with a band-pass filter of 50–1,000 Hz.

#### Perturbation: muscle responses and trunk admittance.

Small-amplitude trunk perturbations were delivered to the trunk in the anterior direction at the T_10_ level by a magnetically driven linear actuator (GD250XS; NiLAB GmbH, Germany) with a stroke length of 0.7 m, controlled by a servodrive (Xenus XTL; Copley Controls, United States) as employed in previous studies (50, 52, 53). To help maintain contact between the rod and the participant, a patch (5 × 5 cm) made of thermoplastic material was shaped to attach to the participant’s back at the appropriate level (Fig. 2). The linear actuator was controlled with custom-made software (LabVIEW 2017; National Instruments, United States) via a real-time data control and acquisition system (NI 9063; National Instruments, United States). The linear actuator recorded rod position with Hall effect sensors via a digital input module (NI 9411; National Instruments, United States). A subminiature load cell (LCM201-100N; Omega, United Kingdom) was attached to the tip of the rod to measure contact force between the rod and participant, with the signal conditioned by a bridge module (NI 9237; National Instruments, United States) within the data acquisition system. The load cell force signal was filtered with a fourth-order low-pass Butterworth filter with a cutoff frequency of 400 Hz.

**Fig. 3.**
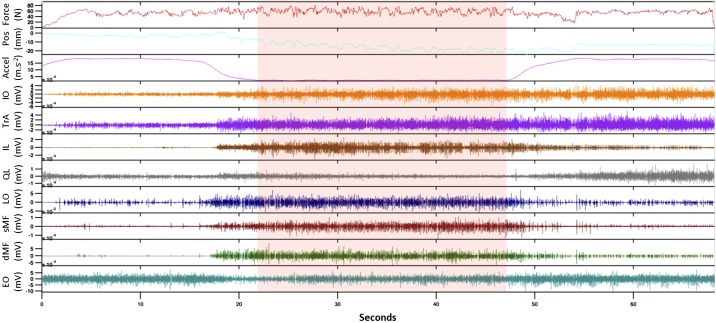
Raw traces recorded during perturbation from a representative participant and parabola (0.5 g). The target force (Force, N), position (Pos, mm), axial acceleration (Accel, m·s^−2^), and intramuscular electromyography (iEMG, mV) of 8 muscles are displayed in the time domain (seconds). The time window of interest (shaded area) was manually selected from the period when the axial acceleration was stable at the corresponding target gravity level. Trunk perturbations started with a 3-s ramp force increase to 60 N of preload during the pull-up phase and terminated at the end of the pull-out phase of each parabola. IO, internal oblique; TrA, transversus abdominis; IL, iliocostalis; QL, quadratus lumborum; LO, longissimus; sMF, superficial multifidus; dMF, deep multifidus; EO, external oblique.

**Fig. 4.**
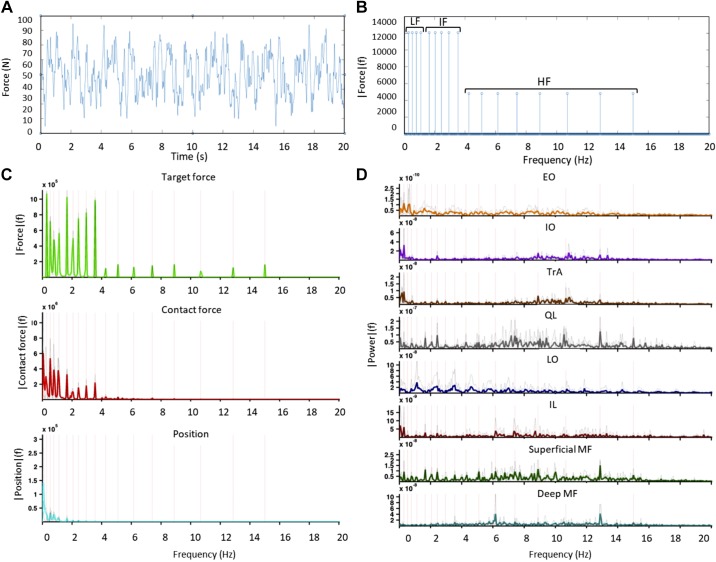
*A*: time domain representation of the actuator’s input, showing the target force applied to the participant. *B*: Fourier transform of the actuator’s input, indicating the low frequency (LF), intermediate frequency (IF), and high frequency (HF) bands. *C*: Fourier transform of the target force, contact force, and actuator position calculated over a selected time window during perturbation. *D*: Fourier transform of the intramuscular electromyography (iEMG) of each muscle during the same time window. EO, external oblique; IO, internal oblique; TrA, transversus abdominis; QL, quadratus lumborum; LO, longissimus; IL, iliocostalis; MF, multifidus.

During trunk perturbations, participants were instructed to resist the perturbation, thus minimizing flexion/extension excursion of the trunk (52). The trunk perturbation started with a 3-s ramp force increase to 60 N of preload, designed to maintain contact with the participant’s back. A dynamic disturbance (±35 N) was then superimposed to the preload (Fig. 3). The dynamic disturbance comprised a crested multisine of 10-s duration, containing 17 logarithmically spaced frequency peaks with a bandwidth ranging from 0.3 to 15 Hz (Fig. 4). The superimposed force was delivered pseudorandomly to avoid voluntary activation on the perturbation (52). The actuator’s input (target force) and position were sampled at 400 Hz and recorded with the actual contact force.

#### Data collection and processing.

The acceleration acting on the participant (i.e., the resultant vector sum between the Earth’s gravitational force and the aircraft acceleration) was recorded with a three-axis accelerometer (Blue Thunder; Vicon IMeasureU Ltd., Auckland, New Zealand) fixed on an experimental rack. iEMG and kinematic data were triggered by a digital signal sent from the real-time control of the LabVIEW software for synchronization. For each parabola, the time window used for analysis was manually selected when the axial acceleration was stable at the corresponding target gravity level (Fig. 4).

During rest, spinal curvature in the sagittal plane was estimated by calculating the angle between the two corresponding spinal segments: cervico-thoracic curvature (C_7_–T_3_ vs. T_3_–T_7_), thoracic kyphosis (T_3_–T_7_ vs. T_7_–T_12_), thoraco-lumbar curvature (T_7_–T_12_ vs. T_12_–L_3_), and lumbar lordosis (T_12_–L_3_ vs. L_3_–S_1_).

The amplitude of muscle activity during both rest and perturbation was assessed as the root mean square (RMS) of each iEMG signal with an epoch length of 500 ms. To compensate for intersubject variability in iEMG amplitude and to enable comparison of amplitude between conditions (within subject), the RMS iEMG from each trial was normalized to the peak RMS iEMG recorded across the trials at 0.75 g. This condition was chosen as reference because it most likely includes the highest levels of activation of the trunk muscles across conditions and thus avoided potential inconsistencies in normalization using smaller values.

With an algorithm developed and validated previously (52), closed-loop system identification was used to estimate the trunk admittance (the inverse of trunk stiffness) and the muscle responses as frequency-response functions (FRFs) from the perturbation. The trunk admittance describes the actuator displacement (x_A_) as a function of contact force (F_C_). The muscle response describes the EMG amplitude of each muscle (EMG*_j_*) as a function of the actuator displacement. Both were evaluated in the frequency domain at the frequencies contained in the perturbation (F_Pert_) signal (Fig. 4).

$${\hat{H}}_{\text{adm}}\left(f\right)=\frac{{\hat{S}}_{{\text{F}}_{\text{Pert}}{\text{x}}_{\text{A}}}\left(f\right)}{{\hat{S}}_{{\text{F}}_{\text{Pert}}{\text{F}}_{\text{C}}}\left(f\right)}\text{; }{\hat{H}}_{{\text{EMG}}_{j}}\left(f\right)=\frac{{\hat{S}}_{{\text{F}}_{\text{Pert}}{\text{EMG}}_{j}}\left(f\right)}{{\hat{S}}_{{\text{F}}_{\text{Pert}}{\text{x}}_{\text{A}}}\left(f\right)}$$

with ${\hat{S}}_{{\text{F}}_{\text{Pert}}{\text{x}}_{\text{A}}}\left(f\right)$ representing the cross-spectral density between signals F_Pert_ and x_A_, and so on. The corresponding coherence functions associated with the admittance and each EMG response were also calculated as described previously (52).

$${\hat{\gamma }}_{\text{adm}}^{2}\left(f\right)=\frac{{\left|{\hat{S}}_{{\text{F}}_{\text{Pert}}{\text{x}}_{\text{A}}}\left(f\right)\right|}^{2}}{{\hat{S}}_{{\text{F}}_{\text{Pert}}{\text{F}}_{\text{Pert}}}\left(f\right){\hat{S}}_{{\text{x}}_{\text{A}}{\text{x}}_{\text{A}}}\left(f\right)}\text{; }{\hat{\gamma }}_{{\text{EMG}}_{j}}^{2}\left(f\right)=\frac{{\left|{\hat{S}}_{{\text{F}}_{\text{Pert}}{\text{EMG}}_{j}}\left(f\right)\right|}^{2}}{{\hat{S}}_{{\text{F}}_{\text{Pert}}{\text{F}}_{\text{Pert}}}\left(f\right){\hat{S}}_{{\text{EMG}}_{j}{\text{EMG}}_{j}}\left(f\right)}$$

The coherence function evaluates the frequency-dependent input-output correlation and can attain values from 0 to 1, where 1 reflects a perfect, noise-free association. To improve the accuracy in the estimation of these frequency parameters, signals were divided in sections of 10 s, each containing all the frequency components in the perturbation protocol, and then averaged in the frequency domain. The number of sections varied across gravity levels according to the duration of the parabolas (see Fig. 1). The results of the FRF gains and coherence were averaged across frequency bands, corresponding to low (0.30–1.10 Hz), intermediate (1.65–3.55 Hz), and high (4.25–15.00 Hz) frequencies, which are believed to represent different trunk-stabilizing mechanisms (for more details see Ref. 52): the low-frequency response reflects intrinsic stiffness and position feedback; the intermediate frequencies are dominated by intrinsic damping and velocity feedback; and high frequencies are influenced by trunk mass, force, and/or acceleration feedback (52).

Finally, to evaluate whether breathing interfered with the abdominal and trunk muscle activity during the perturbation, the EMG power at the main breathing frequency (which is generally ~0.2 Hz) was expressed as a percentage of the total EMG power at frequencies between 0 and 2 Hz (1).

#### Statistical analysis.

Normality of data was assessed with the Shapiro–Wilk test. Normality having been established, one-way repeated-measures ANOVAs were performed to compare spinal curvatures at rest, the RMS iEMG, RMS iEMG power at 0.2 Hz, and FRF gains from each frequency band, between gravity levels (0.25 g, 0.5 g, and 0.75 g; within-subject factor). Where appropriate, post hoc analyses were performed using a Bonferroni multiple comparison test to identify when gravity levels differed.

Repeated-measures correlation was used (with the R function “rmcorr”) to assess the association between the mean axial acceleration and the RMS EMG, spinal angles, EMG power at 0.2 Hz, and FRF gains at each frequency band (4). Because of multiple correlation analyses with the mean axial acceleration, significance level was corrected by the number of muscles (0.05/8; *P* < 0.00625) for RMS EMG, EMG power at 0.2 Hz, and FRF gains at each frequency band or the number of angles (0.05/4; *P* < 0.0125) for the spinal angles.

All data are presented as means ± standard deviation (SD), unless otherwise indicated. Statistical analyses were performed with Stata (v14.0) for the one-way repeated-measures ANOVA and R (R-3.6.1) for the repeated-measures correlation (function “rmcorr” not available in Stata). Significance level was set at *P* < 0.05, and corrections for repeated measures were applied when relevant.

## RESULTS

### 

#### Participants and axial acceleration.

The scopolamine hydrobromide, fine wire insertion, and parabolic flight were well tolerated by all participants without any adverse effects. The iEMG data from the deep MF muscle of one subject were excluded because of excessive noise in the recordings.

The average axial accelerations (perpendicular to the long axis of the aircraft fuselage) during the parabolas, recorded by the accelerometer placed on the device frame, were 2.34 ± 0.02 m·s^−2^ (i.e., 0.239 g), 4.86 ± 0.06 m·s^−2^ (i.e., 0.495 g), and 7.33 ± 0.02 m·s^−2^ (i.e., 0.747 g) during 0.25 g, 0.5 g, and 0.75 g, respectively.

#### RMS EMG and spinal curvatures during rest.

A significant effect of gravity level was found for the LO muscle RMS EMG (*F*_2,10_ = 11.43; *P* = 0.003). Post hoc testing demonstrated that LO RMS EMG was 52.0 ± 22.4% lower at 0.25 g than at 0.75 g (*P* = 0.002). No significant differences were found between 0.25 g and 0.5 g (*P* = 0.12) or between 0.5 g and 0.75 g (*P* = 0.11). There was no significant effect of gravity level on the RMS EMG of the other trunk muscles (*F*_2,10_ all < 2.15; *P* > 0.17) or the spinal angles (*F*_2,10_ all < 0.60; *P* > 0.56; Table 1).

**Table 1.** Spinal angles averaged across all subjects

Spinal Angles	0.25 g	0.50 g	0.75 g	*F*_2,10_	*P*
C_7_–T_3_ vs. T_3_–T_7_	15.3 ± 6.2	15.7 ± 5.1	15.7 ± 4.8	0.17	0.846
T_3_–T_7_ vs. T_3_–T_12_	18.8 ± 4.1	18.6 ± 4.3	18.0 ± 4.7	0.58	0.577
T_7_–T_12_ vs. T_12_–L_3_	5.6 ± 6.4	5.5 ± 7.0	4.9 ± 6.5	0.60	0.568
T_12_–L_3_ vs. L_3_–S1	−4.7 ± 4.0	−4.9 ± 4.5	−4.7 ± 3.7	0.06	0.939

Values are means ± SD. One-way repeated-measures ANOVAs were used to compare spinal angles between gravity levels (0.25 g, 0.5 g, and 0.75 g).

#### RMS EMG and power at 0.2 Hz (respiratory frequency) during perturbation.

A significant effect of gravity level was found for the TrA RMS EMG (*F*_2,10_ = 9.91; *P* = 0.005). Post hoc testing demonstrated that TrA RMS EMG was 60.8 ± 43.8% greater at 0.25 g than at 0.75 g (*P* = 0.005). No significant differences were found between 0.25 g and 0.5 g (*P* = 0.22) or between 0.5 g and 0.75 g (*P* = 0.19). There was no significant effect of gravity level on the RMS EMG of the other trunk muscles (*F*_2,10_ all < 2.15; *P* > 0.17) or the EMG power of any muscle at 0.2 Hz (*F*_2,10_ all < 3.27; *P* > 0.081).

#### Frequency-response functions during perturbation.

**Fig. 5.**
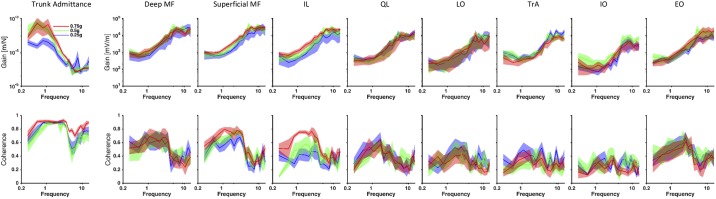
The average ± SE across subjects of the frequency-response functions, i.e., trunk admittance and EMG responses (*top*), as well as the corresponding coherence functions (*bottom*). The average values and the SE across subjects can be observed at each frequency measured, corresponding to the peak frequencies of the perturbation signal delivered by the actuator. MF, multifidus; IL, iliocostalis; QL, quadratus lumborum; LO, longissimus; TrA, transversus abdominis; IO, internal oblique; EO, external oblique.

The frequency-response functions (FRFs) presented coherence levels ranging from 0.61 ± 0.16 to 0.91 ± 0.02 for trunk admittance and from 0.19 ± 0.09 to 0.76 ± 0.02 for muscle responses (Table 2). Based on the number of disjoint sections used for each condition, the significance threshold for coherence in each gravity level was 0.45 at 0.25 g, 0.31 at 0.5 g, and 0.24 at 0.75 g. The significance threshold for coherence was higher for the lower gravity levels because the parabolas were shorter and hence less data were available for the analysis. In general, the coherence levels of the abdominal muscles were lower than those for the back muscles because of lower myoelectric activity during the task (Fig. 5).

**Table 2.** Coherence between trunk admittance and trunk muscle responses at each of the frequency bands of interest

	Low Frequency (0.30–1.10 Hz)			Intermediate Frequency (1.65–3.55 Hz)			High Frequency (4.25–15.00 Hz)		
	0.25 g	0.50 g	0.75 g	0.25 g	0.50 g	0.75 g	0.25 g	0.50 g	0.75 g
Admittance	0.85 ± 0.08	0.75 ± 0.15	0.86 ± 0.08	0.91 ± 0.04	0.85 ± 0.07	0.91 ± 0.05	0.75 ± 0.12	0.61 ± 0.16	0.80 ± 0.07
Superficial MF	0.54 ± 0.13	0.56 ± 0.06	0.68 ± 0.13	0.71 ± 0.12	0.72 ± 0.06	0.74 ± 0.14	0.42 ± 0.09	0.36 ± 0.13	0.40 ± 0.15
Deep MF	0.64 ± 0.25	0.63 ± 0.21	0.67 ± 0.13	0.75 ± 0.17	0.70 ± 0.19	0.76 ± 0.05	0.44 ± 0.14	0.42 ± 0.14	0.38 ± 0.15
IL	0.41 ± 0.22	0.35 ± 0.16	0.61 ± 0.14	0.55 ± 0.27	0.55 ± 0.27	0.72 ± 0.11	0.40 ± 0.15	0.35 ± 0.17	0.41 ± 0.04
LO	0.37 ± 0.16	0.29 ± 0.21	0.32 ± 0.25	0.59 ± 0.21	0.45 ± 0.31	0.40 ± 0.31	0.39 ± 0.13	0.28 ± 0.17	0.24 ± 0.14
QL	0.48 ± 0.20	0.37 ± 0.09	0.41 ± 0.20	0.57 ± 0.24	0.48 ± 0.23	0.45 ± 0.22	0.37 ± 0.17	0.33 ± 0.18	0.28 ± 0.18
TrA	0.38 ± 0.18	0.23 ± 0.09	0.28 ± 0.12	0.50 ± 0.21	0.34 ± 0.16	0.34 ± 0.20	0.35 ± 0.12	0.25 ± 0.16	0.25 ± 0.15
OI	0.29 ± 0.10	0.28 ± 0.17	0.21 ± 0.15	0.35 ± 0.10	0.32 ± 0.24	0.27 ± 0.14	0.31 ± 0.08	0.29 ± 0.16	0.19 ± 0.09
OE	0.45 ± 0.27	0.48 ± 0.26	0.40 ± 0.28	0.62 ± 0.23	0.55 ± 0.28	0.56 ± 0.27	0.40 ± 0.16	0.39 ± 0.22	0.31 ± 0.19

Values are mean ± SD coherence between trunk admittance and trunk muscle responses at each of the frequency bands of interest: low (0.30–1.10 Hz), intermediate (1.65–3.55 Hz), and high (4.25–15.00 Hz). Coherence measures were averaged across participants. IL, iliocostalis lumborum; LO, longissimus thoracis; MF, multifidus; OE, obliquus externus; OI, obliquus internus; QL, quadratus lumborum; TrA, transversus abdominis.

**Fig. 6.**
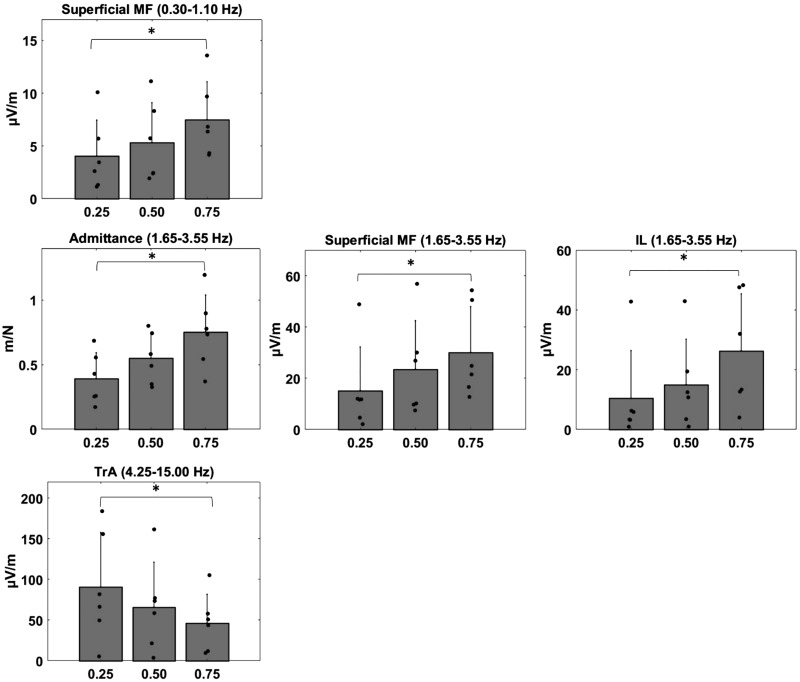
Trunk admittance and EMG responses that were found to have significant differences between gravity levels. Values represent the group mean (bars) and standard deviation (error bars). MF, multifidus; IL, iliocostalis; TrA, transversus abdominis. *Post hoc analysis gravity effect (*P* < 0.05).

For the gains at the low frequencies (0.3–1.10 Hz), a significant effect of gravity level was found for superficial MF responses (*F*_2,10_ = 4.46; *P* = 0.041) (Table 3). Post hoc tests revealed lower superficial MF response gains at 0.25 g than at 0.75 g (*P* = 0.043) (Fig. 6). No significant differences were found between 0.25 g and 0.5 g (*P* = 0.67) or between 0.5 g and 0.75 g (*P* = 0.29).

**Table 3.** Amplitude of trunk admittance and muscle responses at low frequencies (0.30–1.10 Hz), averaged across all subjects

	Low Frequencies (0.30–1.10 Hz)			One-Way Repeated-Measures ANOVA	*P* Value
	0.25 g	0.50 g	0.75 g		
Admittance, m/N	0.91 ± 0.46	2.40 ± 1.24	2.46 ± 2.05	*F*_2,10_ = 2.08	0.176
Superficial MF, µV/m	4.01 ± 3.27	5.32 ± 3.77	7.48 ± 3.59	*F*_2,10_ = 4.46	**0.041**
Deep MF, µV/m	4.15 ± 4.51	6.38 ± 6.07	6.71 ± 5.77	*F*_2,8_ = 2.67	0.129
IL, µV/m	2.38 ± 2.66	3.57 ± 3.01	6.42 ± 5.00	*F*_2,10_ = 3.72	0.062
LO, µV/m	1.53 ± 2.47	1.54 ± 2.58	1.85 ± 3.45	*F*_2,10_ = 0.65	0.543
QL, µV/m	2.67 ± 1.60	2.18 ± 2.21	2.18 ± 2.91	*F*_2,10_ = 0.45	0.652
TrA, µV/m	3.12 ± 2.37	2.97 ± 3.07	1.83 ± 1.70	*F*_2,10_ = 2.18	0.164
OI, µV/m	0.55 ± 0.37	1.58 ± 2.57	0.77 ± 1.21	*F*_2,10_ = 1.19	0.344
OE, µV/m	2.45 ± 2.11	2.88 ± 3.09	2.70 ± 2.82	*F*_2,10_ = 0.12	0.885

Values are mean ± SD amplitude of trunk admittance and muscle responses at low frequencies (0.30–1.10 Hz) averaged across all subjects. One way repeated-measures ANOVAs were used to compare admittance and muscle responses between gravity levels (0.25 g, 0.5 g, and 0.75 g). Boldface indicates *P* < 0.05. IL, iliocostalis lumborum; LO, longissimus thoracis; OE, obliquus externus; OI, obliquus internus; MF, multifidus; QL, quadratus lumborum; TrA, transversus abdominis.

For the gains at the intermediate frequencies (1.65–3.45 Hz), a significant effect of gravity was found for the trunk admittance (*F*_2,10_ = 4.31; *P* = 0.045) and for the muscle response gain of superficial MF (*F*_2,10_ = 5.14; *P* = 0.029) and IL (*F*_2,10_ = 4.65; *P* = 0.037) (Table 4). Post hoc tests revealed lower trunk admittance (*P* = 0.045) and lower superficial MF and IL muscle response gains (*P* = 0.029 and *P* = 0.043, respectively) at 0.25 g than at 0.75 g (Fig. 6). No significant differences were found between 0.25 g and 0.5 g (all *P* > 0.31) or between 0.5 g and 0.75 g (all *P* > 0.18).

**Table 4.** Amplitude of trunk admittance and muscle responses at intermediate frequencies (1.65–3.55 Hz) averaged across all subjects

	Intermediate Frequencies (1.65–3.55 Hz)			One-Way Repeated-Measures ANOVA	*P* Value
	0.25 g	0.50 g	0.75 g		
Admittance, m/N	0.39 ± 0.19	0.55 ± 0.20	0.75 ± 0.28	*F*_2,10_ = 4.31	**0.045**
Superficial MF, µV/m	15.17 ± 17.03	23.52 ± 18.93	30.08 ± 17.83	*F*_2,10_ = 5.14	**0.029**
Deep MF, µV/m	19.62 ± 28.40	26.31 ± 31.11	25.46 ± 27.70	*F*_2,8_ = 3.83	0.068
IL, µV/m	10.41 ± 15.98	15.00 ± 15.18	26.32 ± 19.07	*F*_2,10_ = 4.65	**0.037**
LO, µV/m	3.54 ± 5.59	5.62 ± 9.94	5.59 ± 10.10	*F*_2,10_ = 1.22	0.335
QL, µV/m	10.41 ± 13.81	9.83 ± 16.65	8.74 ± 15.50	*F*_2,10_ = 0.66	0.539
TrA, µV/m	5.77 ± 3.76	4.45 ± 2.92	3.98 ± 3.59	*F*_2,10_ = 2.43	0.138
OI, µV/m	1.16 ± 1.22	2.73 ± 2.46	2.14 ± 2.72	*F*_2,10_ = 1.35	0.303
OE, µV/m	6.61 ± 7.10	7.83 ± 7.82	8.39 ± 8.63	*F*_2,10_ = 0.29	0.757

Values are mean ± SD amplitude of trunk admittance and muscle responses at intermediate frequencies (1.65–3.55 Hz) averaged across all subjects. One way repeated-measures ANOVAs were used to compare admittance and muscle responses between gravity levels (0.25 g, 0.5 g, and 0.75 g). Boldface indicates *P* < 0.05. IL, iliocostalis lumborum; LO, longissimus thoracis; OE, obliquus externus; OI, obliquus internus; MF, multifidus; QL, quadratus lumborum; TrA, transversus abdominis.

For the gains at the high frequencies (4.25–15.00 Hz), a significant effect of gravity level was found for the TrA response (*F*_2,10_ = 4.93; *P* = 0.034) (Table 5). Post hoc tests showed greater TrA muscle response gains at 0.25 g than at 0.75 g (*P* = 0.032) (Fig. 6). No significant differences were found between 0.25 g and 0.5 g (*P* = 0.33) or between 0.5 g and 0.75 g (*P* = 0.58).

**Table 5.** Amplitude of trunk admittance and muscle responses at high frequencies (4.25–15.00 Hz) averaged across all subjects

	High Frequencies (4.25–15.00 Hz)			One-Way Repeated-Measures ANOVA	*P*
	0.25 g	0.50 g	0.75 g		
Admittance, m/N	0.34 ± 0.29	0.27 ± 0.08	0.28 ± 0.03	*F*_2,10_ = 0.36	0.703
Superficial MF, µV/m	147.91 ± 192.00	208.77 ± 148.93	206.85 ± 139.69	*F*_2,10_ = 0.68	0.528
Deep MF, µV/m	158.55 ± 239.31	185.51 ± 177.48	146.10 ± 148.06	*F*_2,8_ = 0.38	0.695
IL, µV/m	82.92 ± 125.31	145.09 ± 170.09	157.33 ± 90.21	*F*_2,10_ = 0.86	0.451
LO, µV/m	54.74 ± 102.90	53.53 ± 89.12	42.84 ± 80.11	*F*_2,10_ = 1.03	0.393
QL, µV/m	78.98 ± 78.07	73.05 ± 73.30	71.70 ± 98.31	*F*_2,10_ = 0.39	0.684
TrA, µV/m	90.51 ± 67.17	65.99 ± 55.19	46.58 ± 35.11	*F*_2,10_ = 4.93	**0.032**
OI, µV/m	24.11 ± 33.66	44.61 ± 50.12	20.26 ± 24.48	*F*_2,10_ = 1.11	0.366
OE, µV/m	49.37 ± 48.29	79.45 ± 87.13	45.78 ± 35.73	*F*_2,10_ = 0.54	0.601

Values are mean ± SD amplitude of trunk admittance and muscle responses at high frequencies (4.25–15.00 Hz) averaged across all subjects. One way repeated-measures ANOVAs were used to compare admittance and muscle responses between gravity levels (0.25 g, 0.5 g, and 0.75 g). Boldface indicates *P* < 0.05. IL, iliocostalis lumborum; LO, longissimus thoracis; OE, obliquus externus; OI, obliquus internus; MF, multifidus; QL, quadratus lumborum; TrA, transversus abdominis.

#### Correlation between spinal angles, muscle activity, and axial acceleration during rest and perturbation.

**Fig. 7.**
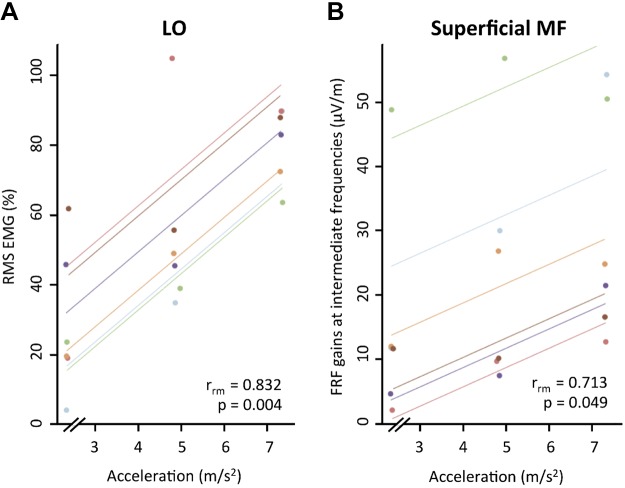
Each dot represents the root mean square electromyography (RMS EMG) of LO (longissimus) during rest and the axial acceleration (*A*) and the muscle responses at the intermediate frequencies (1.65–3.55 Hz) of superficial multifidus (MF) during trunk perturbation and the axial acceleration (*B*). Color identifies participant, and colored lines show repeated-measures correlation fits for each participant.

A significant correlation was found between the RMS EMG of LO muscle during resting state and the axial acceleration [repeated-measures correlation (*r*_rm_) = 0.83; *P* = 0.004] (Fig. 7*A*) and between the gains at the intermediate frequencies of superficial MF and the axial acceleration (*r*_rm_ = 0.71; *P* = 0.049) (Fig. 7*B*). No significant correlations were found between the axial acceleration and any other parameter assessed: spinal angles, RMS EMG at rest state or perturbation, EMG power at 0.2 Hz, or the FRF gains for each frequency band (all *r*_rm_ < 0.70; *P* > 0.054).

## DISCUSSION

This study demonstrates for the first time the immediate effects of hypogravity on trunk stabilization and spinal muscle responses. In comparison to the highest hypogravity condition (0.75 g), low hypogravity (0.25 g) induced *1*) lower myoelectric activity of the LO muscle at rest; *2*) lower trunk admittance and lumbar muscle (superficial MF, IL) response during perturbation; and *3*) greater TrA muscle responses during perturbation. Taken together, these findings suggest that exposure to hypogravity reduces the neuromuscular contribution of (antigravity) trunk extensor muscles to control spinal posture at rest (LO) and during perturbation (IL and superficial MF), with a concomitant increase in deep abdominal muscle (TrA) responses to perturbations.

### 

#### Spinal curvature and neuromuscular control of the spine at rest.

We hypothesized that the lumbar lordosis would become more flattened and that the activity of the trunk muscles required to maintain the lumbar lordosis in an upright posture, such as the thoracic and lumbar erector spinae and the multifidus, would reduce when exposed to lower gravity levels compared with higher gravity levels. Our data did not show any change in spinal posture across conditions, and only the muscle activity of the longissimus thoracis muscle was less in low than high hypogravity. Activity of the deep and superficial multifidus muscles and the lumbar iliocostalis (in sitting) is greatest when the lumbar spine is in a position of lordosis (9), in line with the principal role of the multifidus muscle to produce posterior sagittal rotation of each vertebra, control the lumbar lordosis, and compress the lumbar vertebrae and disks (6). In the present study, participants maintained a relatively flexed rather than lordotic position (around −4.8 ± 4.1°; Table 1), which contrasts with the mean of around −15 ± 10° of lordosis that can be achieved when participants are instructed to sit with a lumbar lordosis and a thoracic kyphosis (9). This most likely decreased the activation of these muscles in the experimental position and may have limited the potential to detect electromyographic changes.

With iEMG electrodes and recording locations similar to those used in the present study, LO EMG of ~10–12% of the maximal voluntary contraction (MVC) has been observed when participants were instructed to sit with a lumbar lordosis and a thoracic kyphosis, but only ~1% MVC was observed when participants assumed a flexed/slumped posture of the thoracic and lumbar spine (9). Tonic activation of LO at ∼8% of MVC is necessary to maintain upright spine posture in sitting in 1 g conditions (9). Those data indicate a relationship between muscle activity and spinal postures to maintain the upright vertebral column against gravity. The reduced LO myoelectric activity observed in the present investigation in lower gravity levels is most likely explained by an adaptation of the muscle to reduced gravity.

#### Neuromuscular control of the spine during perturbation.

Consistent with our hypotheses, exposure to the lower gravity levels induced lower trunk admittance (increased resistance against perturbation) than that recorded at higher gravity levels. That is, the displacement of the trunk by the applied load was less at lower gravity levels, which is explained by reduced destabilizing moments applied to the spine by lower gravity. Gravity destabilizes the trunk by amplifying any displacement resulting from the perturbation. This was despite reduced responses of the superficial MF and IL muscles at 0.25 g, which implies reduced reflexive drive to the paraspinal lumbar muscle. The concomitant increase observed in the muscle response TrA at higher frequencies during 0.25 g suggests increased motor output of this abdominal muscle. In previous studies, activity of the TrA muscle has been shown to contribute to spinal stabilization by increasing intra-abdominal pressure and tensioning of the thoracolumbar fascia (18). However, the mechanical effects of these mechanisms at such high frequencies are unknown and potentially limited because of damping in tissue deformation before force transfer to the spine. Moreover, low values in the coherence function for TrA indicate low reliability of these frequency estimates. Furthermore, although increased TrA response may contribute to decrease admittance, the response of that muscle increased at the higher frequency range, whereas the admittance was lower at the intermediate frequencies.

Several mechanisms may explain the observed reduction of the neuromuscular responses to perturbation of the paraspinal lumbar muscles at lower gravitational loads in the present study. Neuromuscular responses are influenced by a range of sensory inputs from proprioceptors, in addition to the visual and vestibular systems that converge within spinal and supraspinal motor networks (11). Then, from the spinal and supraspinal motor networks, a motor response to the trunk muscles is generated to adjust the torque around the vertebrae (29, 33, 34). As subjects were blindfolded, changes in visual feedback probably contributed little to the changes in neuromuscular responses in the setup used in the present study (31, 51), but the muscle spindle responsiveness has been described to play an important role in sagittal plane trunk stabilization (50, 52).

Micro- and hypogravity affect neuromuscular mechanisms at spinal (36, 38, 44, 47) and supraspinal (10, 49) levels. Consistent with the reduced response observed in some lumbar muscles, previous studies have shown reduced motoneuron excitability in lower limb (“antigravity”) muscles (36, 44). The Hoffman reflex (the electrical analog of the stretch reflex) in the soleus muscle is reduced during hypogravity parabolic flight and excitability of the motoneuron correlated with reduced gravitational forces (44). Although this has been interpreted to suggest gravity-dependent changes in spinal neurons (36, 38, 47), H reflexes cannot exclude the effects of presynaptic inputs on the Ia afferents. This is because the presynaptic inhibition would reduce the response of the motoneuron to the electrically evoked afferent discharge, even when motoneuron excitability is unchanged, and presynaptic inputs are influenced by descending inputs from supraspinal centers (29, 33, 40).

Another possible system involved in the reduction of the muscle responses observed in the present study is the vestibular apparatus. Animal studies provide evidence that muscle spindle discharge is affected by vestibular stimulation (12, 41) via descending inputs to gamma motoneurons (2), which modify the sensitivity of muscle spindles. This effect (increased or decreased excitability) is determined by the pattern of vestibular input (12). The net outcome on alpha motoneuron excitability is also affected by vestibular inputs to the synapse between the Ia afferent from the muscle spindle and the alpha motoneuron, which are depolarized by vestibular inputs. Taken together, these observations could provide an explanation for reduced activity of paraspinal muscle spindles in the present study when gravity is reduced. Although plausible, human studies using galvanic vestibular stimulation to modify vestibular discharge have not observed changes in spindle afferent discharge in leg muscles in awake humans (46). Although it is possible that the effects on muscle spindles of paraspinal muscles, which have some differences in neural pathways (13), might respond differently from leg muscles, available data suggest that direct effects of vestibular stimulation on spindle afferents are unlikely to explain our results.

Functional magnetic resonance imaging (fMRI) has revealed decreased intrinsic connectivity in the right posterior parietal cortex (PPC) immediately after short-term gravitational alterations induced by parabolic flight (49). This cortical area has a role in integration of visual, proprioceptive, and vestibular stimuli (5, 22) and has, via the superior longitudinal fascicle, connections to motor and premotor areas of the cortex (32, 45). Experimental facilitation or inhibition of the PPC by noninvasive cortical stimulation techniques causes transient increases or decreases of the corticomotor excitability and motor behaviors (23, 24). Thus reduced corticomotor excitability secondary to changes in PPC connectivity might explain the reduced paraspinal muscle responses at lower gravity levels as observed in the present study. Although plausible, one study of three participants that evaluated responses to transcranial magnetic stimulation (TMS) over the primary motor cortex during parabolic flights to replicate hypogravity reported a contrasting increased amplitude of motor evoked potentials (MEPs) of the lumbar paraspinal muscles (10). This does not necessarily contradict the results of the present study, as MEP amplitude is determined by excitability of cells in the motor cortex and in the spinal cord, and the effects of each cannot be differentiated (14). Additionally, descending inputs from areas other than the primary motor cortex are likely involved in muscle responses measured with the experimental paradigm used in the present study.

Of note, lower gravitational load reduced the responses of the components of the paraspinal muscles that have capacity to generate extension moments (i.e., LO, IL, superficial MF) and maintain the upright posture of the spine when challenged by gravity. In contrast, there was no significant effect on activation of deep MF in response to perturbation. The deep fibers of the multifidus muscle have a limited moment arm, and therefore can contribute little to spinal extension (6, 37), and may therefore have limited potential to counteract gravity. This might explain why low gravity levels induced changes in responses of superficial MF, LO, and IL but not deep MF. This would suggest a lower, or a slower, impact of long-term exposure to hypogravity on the deep MF.

One possible explanation for the increase in TrA muscle activity with decreasing gravity levels may be an increase in respiratory cycle length. Our data suggest this is unlikely, as the EMG power of the TrA, OI, and OE muscles at 0.2 Hz was not significantly changed.

#### Operational relevance and recommendations for planetary surface explorations.

The results of the present study indicate that short-term exposure to hypogravity reduces paraspinal muscle responses to trunk perturbations and, concurrently, increases the abdominal muscle responses. These findings, and those of previous studies, have shown several sensorimotor adaptations during and after gravitational transitions in parabolic flights [e.g., reduced spinal reflexes (44), decreased intrinsic connectivity in PPC (49), and increased corticomotor excitability of the paraspinal muscles (10)]. If these transient adaptations translate into longer-term effects, it is reasonable to suggest that astronauts would experience modified neuromuscular control of paraspinal and abdominal muscles after long-duration spaceflight. Given the reduction in muscle activity observed at 0.25 g, it is plausible to expect even greater reductions as gravity reduces further (i.e., to 0.16 g and 0 g as present on the lunar surface and in deep space). If this reduction is maintained for long periods of time, capacity of the paraspinal muscles would decline and activity-dependent modification of the trunk muscle behaviors may develop to adapt to a different gravitational condition. This might then contribute to impaired trunk stabilization when reexposed to terrestrial gravity. Although the results of this study suggest that neuromuscular responses are very flexible and can rapidly adapt, prolonged (i.e., weeks or months) activity-dependent modification of the trunk muscle behavior would produce a long-term reduction of muscle function, and the restoration might not occur spontaneously. In view of these possible trunk neuromuscular adaptations, it might be necessary to consider methods to maintain the neuromuscular control of paraspinal and abdominal muscles. For instance, sensorimotor training during predictable or unpredictable trunk perturbations could be applied to maintain constant motor outputs to the paraspinal muscles. In addition, monitoring the activity of the trunk muscles could also be important to tailor specific trunk neuromuscular countermeasures for microgravity (16) before landing on a planetary surface and to prepare for the reintroduction of (hypo) gravity. Finally, the application of artificial gravity or compressive axial loading may mitigate the sensorimotor adaptations provoked by reduced gravity.

#### Limitations.

There are some notable limitations to the present study. First, sample size was small because of the intrinsically complex nature of parabolic flight campaigns and the association limitation to the number of participants that can be studied. Nevertheless, the observation of significant changes in trunk stability and trunk muscle responses despite the small sample size indicates consistent short-term effects of hypogravity in trunk neuromuscular control. However, caution is advised when generalizing results from small populations.

Although analysis of discharge properties of single motor units (MUs) could be considered to disentangle effects of microgravity at an individual MU level, our interest was to understand the overall response of each muscle to the unpredictable force. Previous work has validated this interpretation from analysis of amplitude characteristics using the methods applied in this study (31, 52, 53). The only difference compared with prior studies was the application of several iEMG wires instead of the surface (s)EMG. iEMG electrodes are required to make selective recordings from the small, deep, and multilayered muscles. In these cases conventional sEMG electrodes are inappropriate because of cross talk from adjacent and overlying muscles and signal degradation caused by the nonlinear “volume conductor” effect of physiological tissues (35).

Additionally, fixation of the pelvis and adoption of the kneeling-seated position used in this setup, and the flexed (nonlordotic) lumbar spine positioned position, probably limited the activity of the lumbar paraspinal muscles at rest. It is uncertain whether the results relating to activity of the lumbar muscles and the lumbar lordosis obtained during rest would be comparable with results from participants in standing postures without pelvic fixation.

Finally, the greater response of the trunk extensor (and flexor) muscles is most likely a consequence of the anterior direction of the perturbation applied to the trunk in the sagittal plane. The application of lateral perturbations is likely to affect the trunk muscles differently, potentially with greater responses of the quadratus lumborum, obliquus internus, or externus abdominis muscles, although this needs to be directly tested.

#### Conclusions.

This study reports reduced trunk admittance during perturbation in hypogravity. This reduction was associated with reduced response of the trunk extensor muscles and concomitant increase in transversus abdominis muscle responses. If these motor adaptations were to persist with long-term exposure to hypogravity, they could plausibly have consequences for the control and structure of these muscles. Tailored countermeasures to stimulate the neuromuscular control of trunk and abdominal muscles may be required to reduce the risk for development of modified trunk muscle behaviors after long-term hypo- (micro-) gravity exposures.

## GRANTS

Funding for this study was received from the UK Space Agency and European Space Agency. P. W. H. was supported by a Fellowship (APP1102905) from the National Health and Medical Research Council (NHMRC) of Australia. S. E. S. was funded by a grant (APP1091302) from the NHMRC.

## DISCLOSURES

No conflicts of interest, financial or otherwise, are declared by the authors.

## AUTHOR CONTRIBUTIONS

E.D.M., S.E.S., A.W., K.L., T.W., J.S., D.A.G., J.H., D.D., P.W.H., J.H.v.D., and N.C. conceived and designed research; E.D.M., A.W., K.M., S.R., and N.C. performed experiments; E.D.M. and S.E.S. analyzed data; E.D.M., S.E.S., T.W., P.W.H., J.H.v.D., and N.C. interpreted results of experiments; E.D.M. prepared figures; E.D.M. drafted manuscript; E.D.M., S.E.S., A.W., K.M., K.L., S.R., T.W., J.S., D.A.G., J.H., D.D., P.W.H., J.H.v.D., and N.C. edited and revised manuscript; E.D.M., S.E.S., A.W., K.M., K.L., S.R., T.W., J.S., D.A.G., J.H., D.D., P.W.H., J.H.v.D., and N.C. approved final version of manuscript.
